# Evaluation of RV and LV mass by CMR and comparison to recipient heart following heart transplant; the first-ever CMR human autopsy study

**DOI:** 10.1186/1532-429X-16-S1-P95

**Published:** 2014-01-16

**Authors:** Sahadev T Reddy, Nicholas J Farber, Mark Doyle, Geetha Rayarao, Diane V Thompson, Ronald B Williams, June A Yamrozik, Srinivas Murali, Robert W Biederman

**Affiliations:** 1 Cardiac MRI, Allegheny General Hospital, Pittsburgh, Pennsylvania, USA

## Background

CMR is considered the 'gold standard' for non-invasive LV and RV mass quantitation. To our knowledge, this information is solely based on GRE sequences, with its dependence on flow conditions having never been prospectively or retrospectively validated in humans undermining its credibility. SSFP, with intrinsic contrast dependent on T1/T2 characteristics, offers superior image contrast between blood and myocardium and might redefine the CMR gold standard for cardiac mass. Herein we validate an SSFP approach using explanted hearts obtained from heart transplant recipients.

## Objective

To establish a correlation between SSFP-CMR derived LV and RV mass vs. autopsy mass of *ex vivo *hearts from cardiac transplant patients.

## Methods

Over 3 years, 48 explantations were obtained immediately upon orthotopic heart transplantation from the OR. They were quickly cleaned, prepared (removal of PM/AICDs and/or LV/RVADs), suspended in a saline-filled container and scanned *ex vivo *via SSFP-SA slices definingCMR LV/RV mass (g). Using a readily available automatic thresholding program, segmentation of the slices was achieved in combination with manual trimming (ATMT) of the extraneous tissue using a 3D model by an independent and blinded reader (implemented on a 1.5 GE, Milwaukee, WI). The explanted hearts were dissected with ventricles surgically separated at the inter-ventricular septum. The weight of the total *including *papillary and trabecular myocardium for the LV and RV was measured using a high-fidelity scale. Correlation between the 3D CMR method and pathology were performed along with Bland-Altman plots.

## Results

**Figure 1:**
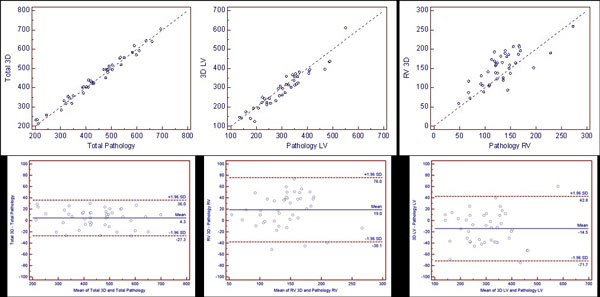
**Top plots - correlation of pathology and CMR**. Lower plots - corresponding Bland-Altman analysis.

With the 48 explanted hearts, 3 (6%) were excluded due to poor image quality (peri-surgical destruction) leaving 45 (94%) for final analysis. Extreme positive correlations were obtained between total CMR 3D mass (448 ± 116 g) and total pathology mass (444 ± 118 g; r = 0.99, p < .001) and the 3D CMR measured LV mass (297 ± 95 g) versus the pathology measured LV mass (308 ± 96 g; r = 0.95, p < .001). Strong positive correlation was demonstrated between the 3D CMR measured RV mass (152 ± 44 g) and pathology measured RV mass (132 ± 41 g; r = 0.76, p < .001). The equation y = 1.01x - 6.6b regressed the LV (r = 0.95). The average bias between 3D CMR and pathology measures for total mass, LV mass and RV mass was: +4.4 g (95% limits of agreement (LOA), -27 to 36 (Figure 1)

## Conclusions

SSFP-CMR accurately determines total myocardial, LV and RV masses as compared to weighed explanted hearts, despite variable surgical removal of instrumentation (LVAD/RVAD, AICDs and often apical core removals). Thus, while GRE was the original 'gold standard' for LV mass, SSFP despite its universal acceptance as the '*de facto *gold standard is now formally validated in a first-ever human autopsy study. Further, the regression equation now includes, not excludes papillary and trabecular mass supporting that our Society should now incorporate both into standard cardiac measurements.

## Funding

Internal.

